# Proportion of Over-The-Counter Medicines Containing a Plant Component and Those with Synthetic Substances Administered among Children in a Bulgarian Population

**DOI:** 10.3390/ph17020192

**Published:** 2024-01-31

**Authors:** Bozhidarka Hadzhieva, Valentina Petkova-Dimitrova

**Affiliations:** 1 Medical College, Medical University of Plovdiv, 15a Vasil Aprilov Blvd., 4000 Plovdiv, Bulgaria; 2 Department of Social Pharmacy, Faculty of Pharmacy, Medical University of Sofia, 2 Dunav St., 1000 Sofia, Bulgaria; vpetkova@pharmfac.mu-sofia.bg

**Keywords:** herbal medicinal products, over-the-counter medicines, children, diseases, childhood age

## Abstract

Over-the-counter medicines are intended to influence a number of symptoms and also to cure some human diseases without having to see the doctor. These medicines are used for self-medication and parents also give them to their children. The following fall within the scope of over-the-counter medicines: analgesics, antipyretics, antihistamines, decongestants, gastroprotectors, anti-cough medicines, and others. Their composition also includes one or a combination of medicinal plants. In addition to synthetic substances, some nonprescription medicines contain plant substances and their derivatives. Medicinal plants and their extracted derivatives are applicable in the therapies of a number of diseases. Considering the fact that over-the-counter medicines can be used among children from birth, the subject of our study is those whose composition includes biologically active plant substances. Within this study, we have established the number of nonprescription medicines containing a plant substance individually or in combination with another substance of the same kind and/or other substances, which have been included in a list published on the website of the Bulgarian Drug Agency. The objective of our study is to present the percentage of OTC medicines containing a plant substance intended to affect the symptoms of upper respiratory tract diseases and pain, which are used among children during different periods of their development. Some of these medicines also contain substances such as antihistamines (pheniramine maleate) and decongestants (pseudoephedrine, phenylephrine hydrochloride, dimetidine) that can cause various unwanted side effects. Considering the aforementioned aspects and also the peculiarities of childhood, we recommend that self-treatment be conducted only after consulting a health specialist.

## 1. Introduction

Various historical sources such as written documents, monuments, and some preserved medicines serve as evidence that medicinal plants have been used since ancient times [1,2].

The use of medicinal plants like *Aloe vera* L., *Senna alexandrina* Mill., *Thymus vulgaris* L., *Foenicumum vulgare* L., *Allium sativum* L., and others in the treatment of a number of diseases was described in the Ebers Papyrus, which was written around 1500 BC and contains about 800 prescriptions and 700 plant types [3]. Some of the oldest civilizations of the Chinese, the Egyptians, the Romans, and the ancient Slavs knew a number of medicinal plants and used them for thousands of years. They passed their experience on to other peoples through their folklore and traditions [4]. The medicinal plants common yarrow (*Achillea millefolium* L.) and *chamomile* (*Matricaria chamomilla* L.), which have been written into the European Pharmacopeia, have been used since the time of the Neanderthals and so have poplar buds (*Populus spec.* L.) [5,6,7].

The knowledge about medicinal plants over the past centuries has become scarce. They have been used on the grounds of the accumulated empirical experience over the centuries. The use of herbal medicines has increased over the past years and nowadays, about 80% of the world population continues to use them as part of primary healthcare [8,9]. According to the World Health Organization (WHO), this trend observed even in developed countries is related to the increase and extension of the scope of use of traditional medicines [10]. Some authors believe that the use of medicinal plants could be examined with reference to the following aspects—phytotherapy, nonprescription medicines, and traditional herbal medicines [11,12].

Herbal treatment/phytotherapy is a method of treatment and prophylaxis of diseases by applying medicinal plants, as in different countries, both traditional medicines and standardized herbal extract are used [9,13]. Herbal treatment is based on the empirical knowledge of medicinal plants, while phytotherapy is a scientifically substantiated medical practice and, thus, is distinguished from the more traditional approaches [14].

In order to classify a plant as a drug, it must contain a biologically active substance in its morphological parts. There are a number of plants whose active compounds and therapeutic properties have been scientifically established as a result of in-depth studies, which allows researchers to regard these plants as medicinal plants [15]. The term “herbal drugs” denotes plants or plant parts that have been converted into phytopharmaceuticals by means of simple processes which include collection, drying, and storage [16].

The medicinal value of such medicinal plants depends on the presence of the biologically active ingredient/s with bioactive properties. Studies have been conducted and a number of biologically active substances/ingredients have been identified in plant materials of land and marine origin [17].

Surveys have been performed worldwide in order to establish the composition of medicinal plants and their efficiency. As a result of these surveys, herbal medicines have been developed and produced in order to be used for various indications and by people of different ages [15]. At present, the extraction of bioactive compounds and the synthesis of their analogs is an effective approach for developing herbal products [18].

The use of medicinal plants among children, which is included in some mothers’ “caring” practices, is based on traditions passed on from generation to generation that guarantee good health and the cure of diseases [19]. It has been stated in the existing scientific literature that pediatricians integrate complementary/alternative medical therapy into their conventional therapy for a number of diseases [20,21].

In addition, pharmacists can recommend a medicine sold without a doctor’s prescription that contains a plant substance that can be applied among children in case of a certain disease. No consultation with a medical specialist is required for nonprescription medicines [22]. They can be purchased without the supervision of a medical specialist. Nonprescription medicines or over-the-counter medicines are those determined by the Food and Drug Administration (FDA) to be safe and effective to be used without a doctor’s prescription [23].

In the European Union, there is a complex regulatory framework for traditional herbal medicinal products. It is based on the principle that the marketing authorization issued by the competent authorities is analogous to that required for the marketing of medicinal products. The requirements and procedures for obtaining such marketing authorization are set out in regulations, directives, and scientific guidelines.

Herbal medicinal products fall within the scope of the amended European Directive 2001/83/EC, which requires that any application for authorization to place a medicinal product on the market must be accompanied by documentation containing data on the results of physicochemical, biological, and microbiological tests, as well as pharmacological, toxicological, and clinical tests of the product, which prove its quality, harmlessness, and efficacy.

The amended Directive 2001/83 EC as Directive 2004/24 EC establishes a simplified registration procedure. This procedure should only be acceptable if the herbal medicinal product can be demonstrated to have a medicinal use of sufficient duration within a community. Medical use outside the community should only be taken into account if the medicinal product has been used within the community for a period of a specified duration [24,25].

Today, the member states of the European Union use this directive as a guideline for registering herbal medicines for self-treatment. Registration of these herbal medicines requires evidence of a well-established tradition and that they are safe enough to be marketed as over-the-counter (OTC) medicines. Herbal medicinal products can only be offered in pharmacies under the supervision of a pharmacist and after receiving registration under a full or simplified procedure depending on their classification, i.e., such as over-the-counter drugs and those that must be prescribed by a healthcare professional [26].

In addition to their use for prophylactic purposes, nonprescription medicines are also included in the treatment of a number of conditions such as headaches, ordinary colds, musculoskeletal pains, allergies, nicotine addiction, acids, and others [27].

The composition of some over-the-counter medicines includes plant substances such as: marshmallow (*Althaea officinalis* L.); common mallow (*Malva sylvestris* L.); ribwort plantain (*Plantago lanceolata* L.); Icelandic moos (*Cetraria islandica* (L.) Ach; etc., which mainly have an emollient effect and soothe irritation in case of inflammation of the upper respiratory tract. For example, on the pharmaceutical market in Bulgaria, there are over-the-counter medicines such as: Mucoplant Spitzwegerich Hustcnsaft 5 g/100 g syrup; Tussavit 7.53 g/7.53 g/100 g syrup; and Expectorans No 5, syrup, whose composition includes some of the aforementioned plant raw materials. Other biologically active substances help to extract the secretion that covers the bronchial walls and bronchioles. Plant raw materials having an expectorant effect include the following: primrose (*Primula veris* L.); licorice (*Glycyrrhiza glabra* L.); senega (*Polygala senega* L.); mullet (*Verbascum phlomoides* L.); and soapwort (*Saponaria officinalis* L.). They mainly contain triterpene saponins. The expectorant medicinal plants include cinnamon (*Cinnamomum zeylanicum* Blume); fennel (*Foeniculum vulgare* Mill.); and ginger (*Zingiber officinale* Roscoe) [28,29].

OTC medicines intended to treat pain among children contain substances such as: paracetamol, acetylsalicylic acid, caffeine, propyphenazone, and codeine phosphate (trade name of the Aceffein and Caffetin Forte tablets). Due to the presence of substances such as caffeine and codeine phosphate as well as acetylsalicylic acid in these and other OTC drugs, they can be applied among children over 12 years of age (according to the brief description of the product).

These medicines are used for self-treatment and thus a number of benefits are obtained both medicinally and economically. We need to point out that self-treatment with over-the-counter medicines may have serious consequences when an inappropriate medicine has been used or improper dosage has been applied [30]. Self-treatment with over-the-counter medicines poses a number of risks, mainly among pediatric and geriatric patients. Over-the-counter medicines have the potential to provide faster access to healthcare, but their improper use poses a number of risks [31]. Incorrect self-diagnosis and usage as well as an improper dosage may lead to unwanted side effects, medicinal interactions, and/or unwanted reactions [32]. Therefore, the role of the pharmacist is very important for reducing the risk of any potential unwanted medicinal reactions and also for improving the patients’ results [33].

The purpose of our study is to establish what percentage of the medicines registered as nonprescription medicines are applied among children and contain a plant substance(s) at the same time. We also aimed to establish the presence or the absence of any dependence between the composition of the over-the-counter medicines and the age of the pediatric patients. We would also like to present medicinal plants and their extracted derivatives that are most commonly contained in medicines used for self-treatment.

## 2. Results

An analysis of the list of over-the-counter medicines made in 2020 has shown that out of 999 medicines in total, 198 are applied among children and contain a plant-based active substance.

As a result of the conducted analysis of the registered over-the-counter medicines containing a plant-based active substance, we have established that these medicines are prescribed to children aged 0+ to 18 years old. Depending on the separate periods of the child’s development, we have grouped the different ages from the table of over-the-counter medicines and have obtained the following result: the over-the-counter medicines containing a plant-based active substance that can be used for breastfeeding babies are 2 in number; those applied in early childhood—21; those applied during the pre-school period—49; those applied during the school period to children aged 7–10 years old—7; those applied to children aged 11–14 years old—100; and those applied in the period of adolescence—19.

**Figure 1:**
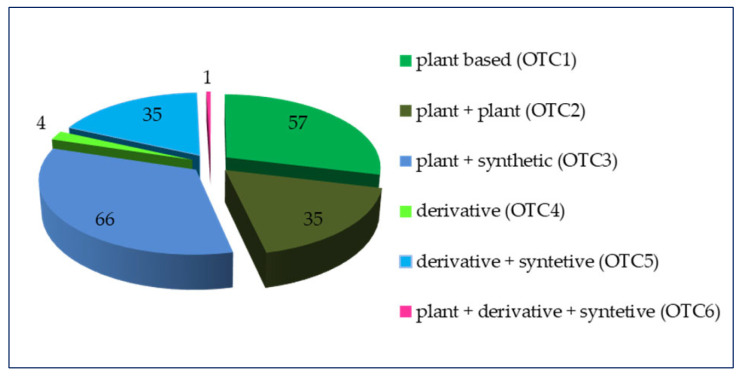
Grouping of plant-based OTCs by active substance.

The grouping based on the content of a plant substance/derivative/synthetic substance of these 198 nonprescription medicines that can be applied among the pediatric population has been illustrated in Figure 1.

Our study has shown that the nonprescription medicines containing the combination of a plant + derivative + syntetive (OTC6) are intended to be applied among children in the secondary school age (Schp, middle). This group is only one in number, owing to which it cannot be expressed in percentage on the graph.

The analyzed medicines are intended to be applied for various diseases, for example, respiratory infections, gastrointestinal problems (acids, meteorism, and others), hepatic diseases (hepatoprotector), pain, edema, trauma, and others.

We have applied statistical analyses to these over-the-counter medicines containing a plant component. We have conducted this study taking into account the most common symptoms typical of childhood, namely, diseases of the upper respiratory tract and pain.

For the purposes of our analysis, we examined each children’s age group as follows: breastfeeding babies by the 28th day (BB by the 28th day); breastfeeding babies by the 12th month (BB by the 12th month); early childhood (Ech); pre-school period (PSchp); school period (Schp), which includes early school age (Schp, early) and secondary school age (Schp, middle); and adolescence (Adul). We grouped the over-the-counter medicines (OTC) containing a plant component in the following way: OTC1—containing only a plant component; OTC2—containing a combination of plant components; OTC3—containing a combination of a plant substance + a synthetic substance; OTC4—containing a derivative; OTC5—containing a combination of a derivative + a synthetic substance; OTC6—containing a combination of a plant substance + a derivative + a synthetic substance. We have excluded OTC4 and OTC6 because with reference to OTC4, there are no such medicines to be applied among children in case of the studied symptoms, and in the OTC6 group, only one medicine has been registered.

### 2.1. Upper Respiratory Tract

**Figure 2:**
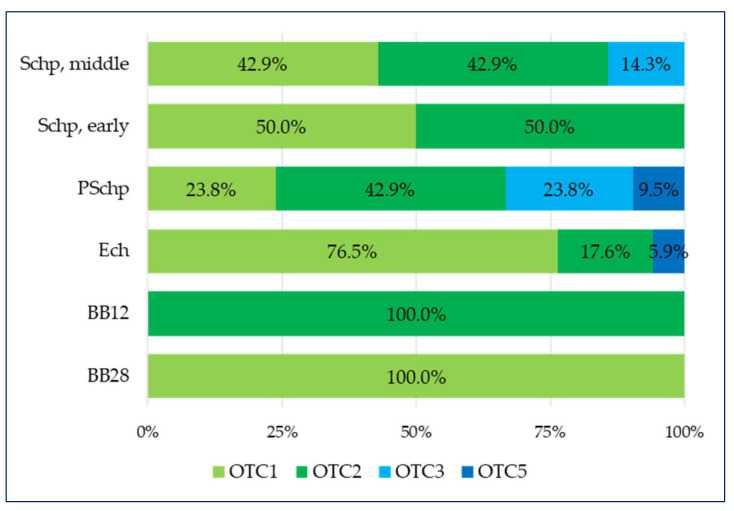
Respiratory tract—distribution of OTCs across age groups.

The two-dimensional division between the “age groups” and “OTC type” variables has shown the following (Figure 2):

- over-the-counter medicines containing only a plant component (OTC1) and over-the-counter medicines containing a combination of a plant substance + a synthetic substance (OTC3) entirely dominate the lowest age groups such as breastfeeding babies by the 28th day (BB28) and breastfeeding babies by the 12th month (BB12);
- three types of OTCs are applied in the Ech group, of which those that dominate are over-the-counter medicines containing only a plant component (OTC1) at 76.5%, and over-the-counter medicines containing a combination of a derivative + a synthetic substance (OTC5) have the smallest share (5.9%);
- in the fourth age group (PSchp), there are four types of OTCs, the largest share (42.9%) of which is over-the-counter medicines containing a combination of a plant components (OTC2) and the smallest share (9.5%) of which is over-the-counter medicines containing a combination of a derivative + a synthetic substance (OTC5);
- in the school period, which includes the early school age (Schp early) group, two types are present—over-the-counter medicines containing only a plant component (OTC1) and over-the-counter medicines containing a combination of a plant components (OTC2), which have equal shares (50% each);
- in the last school period age group that includes the secondary school age (Schp, middle), there are three types of OTC, wherein over-the-counter medicines containing only a plant component (OTC1) and over-the-counter medicines containing a combination of a plant components (OTC2) have equal shares (42.9% each), and the smallest share is comprises over-the-counter medicines containing a combination of a plant substance + a synthetic substance (OTC3) (14.3%).

The analysis has shown that with reference to the diseases of the upper respiratory tract, there are no medicines of types over-the-counter medicines containing a derivative (OTC4) and over-the-counter medicines containing a combination of a plant + a derivative + a syntetive (OTC6).

**Figure 3:**
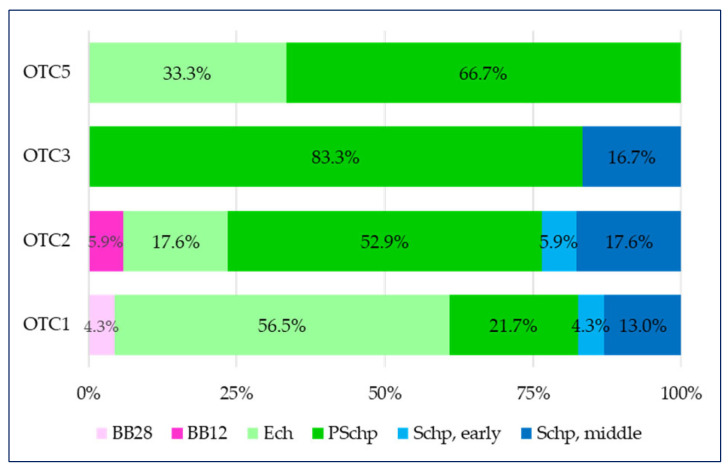
Respiratory tract—share of the age groups within the scope of OTCs.

If we examine the two-dimensional division of medicines from different groups in the aforementioned age groups, we will establish the following (Figure 3):

- in the first group of over-the-counter medicines containing only a plant component (OTC1), there are medicines for five of all age groups (with the exception of breastfeeding babies by the 12th month (BB12)), but over half of them (56.5%) are intended for children in the pre-school period (PSchp);
- there is a similar distribution in the over-the-counter medicines containing a combination of a plant component (OTC2) group where the medicines intended for children in the pre-school period (PSchp) dominate (52.9%), as in this group there are also medicines intended for the youngest children—breastfeeding babies by the 12th month (BB12);
- in the over-the-counter medicines containing a combination of a plant substance + a synthetic substance (OTC3) group, over 80% of all medicines are intended for children in the pre-school period (PSchp) and 16.7% are intended for children in the secondary school period (Schp, middle);
- over-the-counter medicines containing a combination of a derivative + a synthetic substance (OTC5) are divided in a 33% to 66% proportion in two age groups—early childhood (Ech) and pre-school period (PSchp).

On the grounds of the two-dimensional division, an χ^2^ test has been prepared in order to check the presence of any dependence between the two variables. The formulated hypotheses are as follows:

**H_0_**  *There is no dependence between the number of OTCs of different types and the age groups in which they are offered*.

**H_1_**  *We expect some dependence between the number of OTCs of different types and the age groups in which they are offered*.

The result from the check of the zero hypothesis has shown that we have to accept it as being correct.

(Pearson chi-square = 16.284, df = 15, *p* = 0.363).

### 2.2. Pain

**Figure 4:**
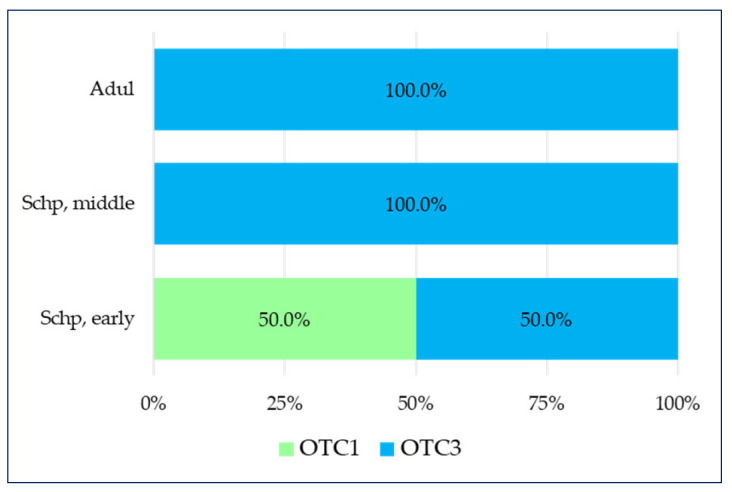
Pain—distribution of OTCs across age groups.

Sixteen medicines in total have been observed in this category as fifteen of them belong to the over-the-counter medicines containing a combination of a plant substance + a synthetic substance (OTC3) group, and one is from the over-the-counter medicines containing only a plant component (OTC1) group. The medicines for pain relief were intended for three age groups—Schp early, Schp middle, and Adul. In two of them (Adul and Schp middle), all of the medicines are from the OTC3 group, whereas in the Schp early group, they are equally divided between OTC1 and OTC3 (Figure 4).

**Figure 5:**
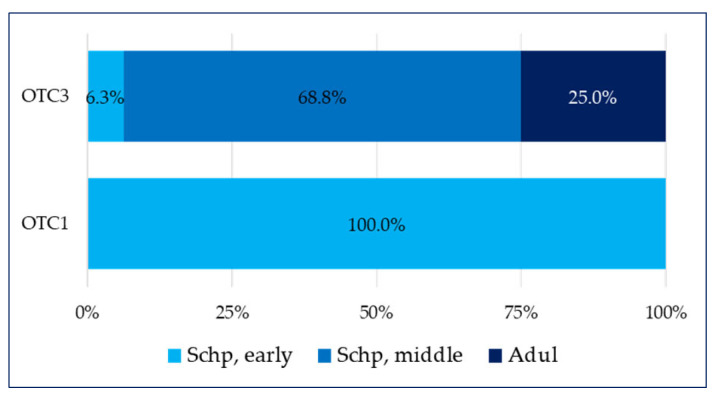
Pain—share of the age groups within the scope of OTCs.

As commented above, OTC1 medicines are entirely intended for the Schp small age group. In the OTC3 group, the division includes a dominant share (68.8%) of the medicines intended for children in the Schp middle group, 25% for children in the Adul group, and 6.3% for the children in the Schp early group (Figure 5).

The development of a child is an incessant process that starts from the child’s birth and continues until the time the child becomes mature. It is characterized by morphological and functional changes of the organs and the systems, maturity of the immune-biological reactivity and improvement of the intellect. Every age in a person’s life is distinguished by its own features and anatomic and physiological peculiarities of adaptation of reactivity, motor activity, and nervous and psychological development.

Considering the fact that the immune system is not completely developed in younger children, children are much more susceptible to viral infections, colds, and influenza [34]. Premature babies are also very susceptible to infections [35]. Children have colds much more frequently, 6–8 times a year, compared to adults who usually have a cold 4–6 times a year [36].

The reasons for a cold among children are different; in most cases, it is caused by viruses, but bacteria can also trigger its emergence. A usual cold lasts for a short time. In most cases, the symptoms reach their peak on the third day and disappear within a week, although the cough may still be present [37]. Nonprescription medicines are also used to influence the cold symptoms, in addition to synthetic substances like paracetamol, dimethidine, and derivatives—pseudoephedrine, and phenylephrine hydrochloride, which are present both individually or in combination—these medicines contain extracts of a number of medicinal plants.

The results from our study have undoubtedly shown that among the youngest pediatric patients, over-the-counter medicines intended to influence the symptoms related to the upper respiratory tract contain mainly a plant component. We have established that among the groups examined by us that are applied to pediatric patients, over-the-counter medicines containing only a plant component (OTC1) and over-the-counter medicines containing a combination of a plant components (OTC2), whose compositions contain plants and their extracted derivatives, are represented mainly by the following plant raw materials: *Tymus vulgaris* L., herba; *Hedera helix* L., folium; *Cetraria islandica* (L.) Ach., tallus; *Althaea officinalis* L., radix or folium; *Primula officinalis* L., radix; and some rare plants such as *Glaucium flavum* Crantz (Glaucine hydrobromide) and *Pelargonium sidoides* DC. Table 1 shows the studied OTC1 and OTC2, with their commercial and international names, their contents of active substances, the symptoms for which they are intended, and the age groups for which they can be applied.

## 3. Discussion

Medicinal plants having a mitigating effect and relieving irritation in case of inflammation of the upper respiratory tract include the following: marshmallow (*Althaea officinalis* L.); common mallow (*Malva sylvestris* L.); ribwort plantain (*Plantago lanceolata L.*); Icelandic moos (*Cetraria islandica* (L.) Ach); coltsfoot (*Tussilago farfara* L.) and others. The effectiveness of (*Althaea officinalis* L.) for coughs has been proven. The blossom of (*Malva sylvestris* L.) contains anthocyanins and mucilage. The mucilage is contained in all parts of the plant and the softening effect on the respiratory tract is due to the presence of this substance [38]. The most important bioactive compounds contained in the leaves of *P. lanceolata* L. are catalpol, aucubin, acteoside, and verbascoside. These compounds are responsible for the anti-inflammatory, antioxidant, antineoplastic, and hepatoprotective effects of the plant [39].

Other traditional plants characterized by mucolytic and antiseptic properties are thyme (*Thymus vulgaris* L.) and garlic (*Allium sativum* L.) [40,41]. The anti-inflammatory effect of extracts of *Tymus vulgaris* L. and *Hedera helix* L. has been studied in a lipopolysaccharide (LPS)-induced model of acute pulmonary inflammation in a rat [42]. The extract of *H. helix* is contained in a number of medicines that can also be applied for children from birth. Its anti-inflammatory, anti-bacterial, antifungal, and anthelmintic properties have been established. A conducted study has described that the simultaneous application of an extract of *H. helix* and Oseltamivir leads to a decrease in the pulmonary inflammation of mice infected with type A influenza virus (PR8). When used perorally, the extract of *H. helix,* whose activity is due to the presence of hederasaponin F, leads to an increased antiviral activity of Oseltamivir, which when applied individually provides insufficient defense against the PRS infection [43].

The medicinal plant *thyme* and its extract are applied perorally for coughs resulting from a cold, bronchitis, laryngitis, and gastrointestinal inflammations, and are also applied locally in the treatment of small wounds and mouth cavity diseases. The plant material contains an ethereal oil whose composition varies depending on the chemotype. The main components of the ethereal oil of *T. vulgaris* L. are: tymol and carvacrol. According to the conducted studies and the scientific sources, these isomeric monoterpenes (tymol, carvacrol) are responsible for the antiseptic, antitussive, and expectorant properties of thyme to a large extent [44].

The stalks of *T. vulgaris* L. and *Tymus zygis* L. are among the most widely used plant materials in the pharmaceutic industry [45]. In the literature, there are descriptions of the activity of cineole and myrtol and also of combined preparations containing primrose, which have manifested good activity during the treatment of sinusitis and bronchitis—some of the most common respiratory infections [46]. Medicines based on medicinal plants are used owing to their antitussive and expectorant effects. Antitussive medicines affect centrally the focus of the cough in the brain or peripherally affect receptors in the respiratory tract.

The use of herbal medicines among children may also pose a number of risks which, on the one hand, arise from the multi-component composition of the plant material and also from the lack of any evidence guaranteeing their safety [47,48]. The continuous use of the so-called traditional plant-based medicines is to be regarded as a guarantee for their safety and efficiency, but their multi-component compositions and the differences in the standardization of various plant materials impede the clinical testing of them. In the composition of the OTC medicines, including those tested by us, there are substances such as paracetamol (acetaminophen), antihistamines (pheniramine maleate), and decongestants (pseudoephedrine, phenylephrine hydrochloride, dimethidine), which lead to a more frequent manifestation of unwanted reactions [49].

Shefrin and Goldman have indicated in their study that the existing evidence related to the application of the over-the-counter medicines in case of infections of the upper respiratory tract is insufficient. In addition, they have also stated that the manifestations of unwanted medicinal reactions resulting from the use of nonprescription medicines are most commonly caused by improper dosage, uncontrolled swallowing, or an inappropriate medicine [50].

Paracetamol, also known as acetaminophen, is a medicine applied individually or in combination with other substances. It has been used since 1955 and is one of the most common medicines for reducing the body temperature (antipyretic) and relieving pain [51]. In medical practice, paracetamol is also a first-line medicine for pain. Based on data contained in the medical literature, paracetamol is safe to be applied from birth and does not cause any hepatic damages when used in accordance with the existing recommendations. Single intakes exceeding the recommended dose ten times are potentially toxic [52]. Conducted studies have described the probability that a chronic use of paracetamol may increase the risk of allergic reactions or asthma among children [53]. The evidence on paracetamol-induced disorders in neurological development is still insufficient [54].

The current study has shown that in the period of breastfeeding (BB12 and BB28) as well as in early childhood (Ech), the composition of the over-the-counter medicines contains primarily substances individually or in combination—as shown in Figure 2. As can be seen from the graph, over-the-counter medicines containing a derivative (OTC4) and OTC6 have not been detected. We could explain their absence with their composition, which comprises a derivative (OTC4) or the combination of a plant + derivative + synthetic substance (OTC6). The presence of a derivative and/or a synthetic substance leads to an increased frequency of the manifestation of unwanted medicinal effects. Another similar study has emphasized that the use of over-the-counter medicines containing antihistamines and decongestants is related to unwanted effects resulting from overdosing and improper use. The American Food and Drug Administration has approved these medicines among adult patients, but with reference to pediatric patients, they have not been tested sufficiently to ensure their safety and efficiency. Owing to this, the FDA has recommended that the content indicated on the label of these medicines be reviewed and also that the use of these medicines among children under 2 years of age be ceased.

Pseudoephedrine and phenylephrine hydrochloride are substances that are present in the composition of medicines used to treat nasal congestion (clogged nose), cold, influenza, and allergies.

Pseudoephedrine (PSE) as a stereoisomer of ephedrine and the medicines containing it, when administered in hid doses, trigger physical symptoms such as reduced appetite, dryness in the mouth, palpitation, and motor symptoms (for example, disruption of gait and balance, postural instability, generalized dystonia, psychomotor retardation, and others) [55]. When applied among pediatric patients, PSE, even in therapeutic doses, may cause the emergence of dizziness, increased heart rate, excitement, and sleeplessness [56,57]. Within the European Union, over-the-counter medicines containing pseudoephedrine can be found on the pharmaceutical market under various tradenames. Owing to considerations related to the safety of the use of medicines containing pseudoephedrine, in early 2023, the Pharmacovigilance Risk Assessment Committee (PRAC) of the European Medicines Agency (EMA) started reviewing these medicines [58].

A study carried out within the period 2003–2006 in Germany has described that self-medication among children and adolescents is increasingly spreading and that this is valid for families with a higher socio-economic status. The use of flu and cold medications, as well as aspirin, among pediatric patients poses potential risks [59]. In general, the rational use of the appropriate OTC drug will result in fewer adverse effects and may be beneficial.

Our study has confirmed that substances like paracetamol, pheniramin maleate, and decongestants are contained in OTCs applied among children in the pre-school and the school periods of their development. The purpose of our subsequent study will be to check N0 again (the zero hypothesis) by comparing the other nosological units in which OTCs containing a plant substance are applied.

## 4. Materials and Methods

### 4.1. A Documentary Analysis of the List of Medicines Registered as Nonprescription Medicines

The list of the over-the-counter medicines was made in 2020 and published on the website of the Bulgarian Drug Agency. The list includes medicines whose permission for use has not expired, although they can be sold until the exhaustion of the available quantity, but no more than a year past the expiry date of the permission for use, which is in accordance with the pharmaceutical legislation of the country.

We have analyzed these medicines based on their active substance(s), indications for use, and age groups. We have distinguished those with a plant component applied among pediatric patients.

### 4.2. Statistical Analysis

Cross-tabulation and chi-square test.

We used the Pearson’s chi-square test (Fisher, 1922; Pearson, 1900) to examine whether there is a relationship between two variables in a cross-table. The purpose of cross-tabulation is to show in a tabular format the relationship between two variables. The purpose of a chi-square test of independence is to determine whether the observed values for the cells deviate significantly from the corresponding expected values for those cells.

Cross-tabulation displays the two variables—age groups (v1) and OTCs (v2)—in a series of rows and columns. The two cross-tables have six rows (v1) and four columns (v2) (v2 has non-zero frequencies for OTC1, OTC2, OTC3, and OTC5). The cross-tabulation table produced using IBM SPSS v.26 contains the number of cases that fall into each combination. We can also look at the percentages within the age groups by looking at the rows. This tells us, for example, that of those within the Ech age group, 76.5% are OTC1, 17.6% are OTC2, and 5.9% are OTC5.

The Pearson’s chi-square (χ^2^) uses the following equation:

$$\chi^{2}=\sum \frac{{\left({observed}_{ij}-{expected}_{ij}\right)}^{2}}{{expected}_{ij}}$$

in which *i* represents the rows in the cross-table and *j* represents the columns.

Expected frequencies for each of the cells in the table are calculated following the equation below:

$$E_{ij}=\frac{{row\;total}_{i}\times {column\;total}_{j}}{n}$$

where *n* is simply the total number of observations.

The $x^{2}\;$ statistic can then be checked against a distribution with known properties. The degrees of freedom of these are calculated as (r − 1)(c − 1), in which r is the number of rows and c is the number of columns. We used SPSS, and this simply produces an estimate of the precise probability of obtaining a chi-square statistic. If the significance value is small enough (conventionally, Sig. must be less than 0.05), then we reject the hypothesis that the variables are independent and gain confidence in the hypothesis that they are in some way related.

Correlation describes the relationship between variables. We assess that relationship (or association) in terms of a ‘correlation coefficient’, which measures the way in which the ‘values’ in one variable (in our case, age groups) change in relation to ‘values’ in a second variable (in our case, OTCs). A positive correlation coefficient occurs when values change in the same direction. A negative correlation exists when values change in opposite directions. We used Spearman’s correlation test (at least one of the variables was not parametric).

During the preparation of the statistical analyses, we used abbreviations for nonprescription medicines and the separate periods of the development of the child/age groups, as indicated in Abbreviations.

## 5. Conclusions

Our study has shown a limited-type diversity of the plant substances contained in nonprescription medicines applied among children. Some nonprescription medicines contain pseudoephedrine, whose safety is currently being examined by the European Drug Agency. As a result, it is recommended that the Bulgarian Drug Agency, in its capacity as a control body for medicines in Bulgaria, reviews the over-the-counter medicines containing pseudoephedrine which are applied among children.

When developing herbal medicines for children, it is necessary to use all of the available resources, integrate the scientific strategies for clinical assessment, and also encourage ethnobotanical studies. Further studying and extending the scope of clinical tests of herbal medicines by applying the latest techniques are necessary, as well as assessments of the bioequivalence of the entire extract. The concept of good clinical practice (GCP) shall focus on the safety and the efficiency of phytopharmaceuticals as a whole and in particular of the phytopharmaceuticals applied among pediatric patients. On the other hand, good pharmacy practice obliges the pharmacist to inform the parents of the rational use of nonprescription medicines and the possible manifestation of unwanted effects and the potential related interactions.

## Figures and Tables

**Table 1:** Registered nonprescription medicines containing plant raw materials announced on the official website of the Bulgarian Drug Agency.

№	Freely Chosen Name	Medicinal Form and Quantity of the Active Substance in a Dose Unit	INN	ATC Code	Content	Use among Pediatric Patients	Plant-Based OTC Depending on the Active Substance	Use of OTC in the Period of (in Accordance with the Age Differentiation among the Pediatric Population)	Therapeutic Indications
1.	Bronchicum	100 mg compressed lozenges × 20; × 50	Thyme fluid extract	R05CA00	Each tablet contains: ✔ *Thymi herba extractum* *siccum* equivalent to 100 mg *Thymi herba extractum* *fluidum* (1: 2–2.5)	Over 4 years of age	OTC1	Pre-school period	Upper respiratory tract
2.	Bronchicum Elixir S	5.0 g/2.5 g/100 g syrup—130 g	Thymi herbae tincture//Grindelii herbae tincture/Quebrachi corticis tincture/Primulae radicis tincture/Pimpinellae radicis tincture	R05CA10	100 mL of the syrup contains: ✔ *Thymus vulgaris/zygis* L., *herba* (1:2–2.5) (liquid extract from a blade of thymes)—5.0 g; ✔ *Primula veris/elatior* L., *radix* (1:2–2.5)—2.5 g.	Over 4 years of age	OTC2	Pre-school period	Upper respiratory tract, cough
3.	Broncholytin	5.75 mg/4.6 mg/5 ml syrup × 125 g	Glaucine bromide/Basilici oil/Ephedrine hydrochloride	R05FA02	B 5 mL of the syrup contains: ✔ *Glaucine hydrobromide*—5.75 mg; ✔ *Eephedrine hydrochloride*—4.6 mg.	Over 3 years of age	OTC2	Pre-school period	Upper respiratory tract, cough
4.	Bronchostop	59.5 mg pastilles × 20; × 40	Plant medicinal product	R05CA	1 pastille contains: 59.5 mg *Thymus vulg./Thymus zyg.* L.) dry extract from blades (7–13:1)	Over 12 years of age	OTC1	School period (middle school age)	Upper respiratory tract, throat
5.	Bronchostop	0.77 g/0.66 g/5 ml—syrup × 150 ml	Plant medicinal product	R05CA10	5 ml (approximately 5.7 g) of syrup contains: ✔ 0.77 g *Thymus vulg./Thymus zyg.* dry extract from blades (1:2–2.5 ✔ *Althaea off*., (liquid extract from roots (1:20)	Over 4 years of age	OTC2	Pre-school period	Upper respiratory tract, cough
6.	Bronchostop Duo	51.1 mg/4.5 mg pastilles × 10; × 20; × 30; × 40	Plant medicinal product	R05CA	1 pastille contains: ✔ 51.1 mg *Thymus vulg./Thymus zyg*.) (7–13:1) water extract); ✔ 4.5 *Althaea off.* (7–9:1)	Over 6 years of age	OTC2	Pre-school period	Upper respiratory tract, cough
7.	Bronchostop Sine	0.12 g/1.28 g/15 ml oral solution—100 ml; × 120 ml; × 150 ml; × 200 ml	Plant medicinal product	R05CA10	15 ml (=15.45 g) of the solution contains: ✔ 0.12 g *Thymus vulg.* *Thymus zyg*. dry extract from blades (7–13:1). ✔ *Althaea off*., liquid extract from the roots (1:12–14)	Over 2 years of age (not recommended for children under 2 years of age)	OTC2	Early childhood	Upper respiratory tract, cough
8.	Bronchoton	4.6 mg/5.75 mg/5 ml syrup × 125 g	Glaucine hydrochloride/Ephedrine hydrochloride/Basilici oil	R05FA02	5 ml of syrup contains: ✔ *Ephedrine hydrochloride*—4.6 mg. ✔ *Glaucine hydrobromide*—5.75 mg.	Over 3 years of age	OTC2	Pre-school period	Upper respiratory tract, cough
9.	Carmolis	Oral drops, solution/cutaneous solution/inhalation vapor, solution—20 ml; × 40 ml, × 80 ml; × 160 ml	Anise oil/Clove oil/Citronella oil/Lavander oil/Lemon oil/Spirit of lemon balm/Menthol racemic/Nutmeg oil/Sage oil/Spiciae aetheroleum/Thyme oil	Plant medicinal product	100 g Carmolis contains: ✔ *Laevomentholum*—1.72000 g. ✔ *Thymi aetheroleum*—0.00172 g; ✔ *Anisi aetheroleum*—0.01548 g; ✔ *Cinn.cassiae aetheroleum*—0.17716 g; ✔ *Caryophylli floris aetheroleum*—0.17716 g; ✔ *Limonis aetheroleum*—0.01118 g; ✔ *Lavandulae aetheroleum*—0.17716 g; ✔ *Spicae aetheroleum*—0.17716 g; ✔ *Citronellae aetheroleum*—0.01720 g; ✔ *Salviae aetheroleum*—0.03526 g; ✔ *Myristicae aetheroleuml* 0.07052 g ✔ *Melissae spiritus*/1:5/	Not recommended for children under 12 years of age	OTC2	School period (middle school age)	Upper respiratory tract, cough
10.	Cefabronchin	50 g/21 g/100 g oral drops, solution × 20 ml, × 50 ml, × 100 ml	Plant medicinal product		100 g of drops contains: ✔ *Thymi herba extractum* *fluidum* (1:2–3) 50 g *Extractum fluidum compositum* (1:3–4) 21 g: ✔ *Lichen islandicus*; ✔ *Saponariae rubrae radix*; ✔ *Pimpinellae radix*; ✔ *Eucalypti folium*; ✔ *Foeniculi amari fructus* (1:1:1:1:1) et 1 pars ✔ *Anisi stellati fructus*	Over 4 years of age	OTC2	Pre-school period	Upper respiratory tract, cough
11.	Hedelix	0.8 g/100 ml syrup	Hederae helicis folium extractum spissum	R05CA12	100 ml of syrup contains: 0.8 g *Hederae helicis folium extractum spissum* with a proportion of the active substance and the extract (2.2–2.9: 1).	Yes 0 +	OTC1	Breastfeeding period	Upper respiratory tract, cough
12.	Hedespan	7 mg/ml syrup ×100 ml	Plant medicinal product	R05CA 0	1 ml contains: 7 mg extr. siccum *Hedera helix* L., *folium*	Over 2 years of age	OTC1	Early childhood	Upper respiratory tract, cough
13.	Herbion iceland Moss	6 mg/ml syrup × 150 ml	Plant medicinal product		1 ml of syrup contains: 6 mg *Cetraria islandica* (L.) Ach, *tallus* (16–18:1)	Yes (for children aged 4–6 after being diagnosed)	OTC1	Pre-school period	Upper respiratory tract, cough
14.	Herbion Ivy	7 mg/ml syrup × 150 ml	Plant medicinal product	R05CA 12	1 ml of syrup contains: 7 mg *Hederae helicis folium* *extractum spirituosum siccum* (5–7.5:1)	Over 2 years of age (contraindicated for children under 2 years of age)	OTC1	Early childhood	Upper respiratory tract, cough
15.	Herbion Ivy	lozengez × 8; × 16; × 24; × 32; × 40	Plant medicinal product	R05CA 12	1 tablet for sucking contains: 35 mg *Hedera helix* L., *folium extractum siccum* (5–7.5:1)	Over 6 years of age	OTC1	Pre-school period	Upper respiratory tract, cough
16.	Hustagil Thyme Cough Syrup	syrup × 150 ml	Thyme fluid extract	R05CA	6 g (= 5 ml) contains medicinal substance: 480 mg *Extr. Thymi fluidum* (DAB) standardized over min. 0.03% Tymol	Over 1 year of age	OTC1	Early childhood	Upper respiratory tract, cough
17.	Ivitus	7 mg/ml oral solution— × 100 ml; × 150 ml; × 200 ml	Expetorants	R05CA 0	1 ml of solution contains: *Hedera helix* L. folium (5–8:1)	Over 2 years of age	OTC1	Early childhood	Upper respiratory tract, cough
18.	Mucohelix	syrup × 100 ml	Hederae helicis folii extractium siccum	R05CA12	1 ml of syrup contains: 8.25 mg *Hederae helicis folium extractum siccum*	Over 2 years of age	OTC1	Early childhood	Upper respiratory tract, cough
19.	Mucoplant Spitzwegerich Hustensaft	syrup 5 g/100 g × 100 ml; × 250 ml	Plant medicinal product	R05FB02	1 g (0.8 ml) of syrup contains: 50 mg *Plantaginis lanceolatae folium extractum fluidum* (*Plantago lanceolata* L.) (1:1)	Over 2 years of age	OTC1	Early childhood	Upper respiratory tract, cough
20.	Mucoplant Anisol	Capsules 100 mg × 30	Anise oil/Anisi aetheroleum	R05CA	1 capsule contains: *Anisi stellati aetheroleum*—100 mg	Over 12 years of age	OTC1	School period (middle school age)	Upper respiratory tract, cough
21.	Mucoplant Cough Syrup Ivy	154 mg/100 ml syrup × 100 ml; × 200 ml; × 250 ml	Ivy leaf dry extract (*Hedera helix* L.) Extraction solvent: Ethanol	R05C	Each ml contains:1.54 mg *Hedera helix* L. (DER 4–8:1)	Over 2 years of age (contraindicated for children under 2 years of age owing to a risk of deterioration of the respective symptoms)	OTC1	Early childhood	Upper respiratory tract, cough
22.	Pinosol	nasal drops, solution 10 ml	Pine oil/Eucalyptus oil/Tocopherol acetate/Thymol/Guaizulene/Peppermint oil	R01AX00	Medicines contain: ✔ *Scots pine, oil*—375.2 mg; ✔ *Hot mint, oil*—100 mg; ✔ *Eucalyptus oil*—50 mg; ✔ *Alpha-tocopheryl acetate*—170 mg; ✔ *Thymol*—3.2 mg; ✔ *Guaiazulene*—2.0 mg. in 10 g solution. 1 mL = 25 gutt	Over 6 years of age for inhalation among children over 12 years of age	OTC2	Pre-school period	Upper respiratory tract, inflammation of the nasal and nasopharyngeal mucous membrane
23.	Prospan	20 mg/ml oral drops, solution × 20 ml	Hedera helix extract	R05FB00	1 ml of solution contains: ✔ 20 mg *Hedera helix folium extractum siccum* (5–7.5:1)	Over 1 year of age	OTC1	Early childhood	Upper respiratory tract, cough
24.	Prospan	65 mg eff. tablets × 10; × 20	Hedera helix extract	R05FB00	1 effervescent tablet contains: 65 mg *Hedera helix folium* *extractum siccum* (5–7.5:1)	Over 12 years of age (children over 6 years of age take ½ of an effervescent tablet 2 times a day)	OTC1	Pre-school period	Upper respiratory tract, cough
25.	Prospan	26 mg lozenges × 10; × 20	Hedera helix extract	R05CA	1 tablet for sucking contains: 26 mg *Hedera helix folium* *extractum siccum* (5–7.5): 1)	Over 6 years of age	OTC1	Pre-school period	Upper respiratory tract, cough
26.	Prospan	7 mg/ml syrup × 5 ml; × 100 ml; × 200 ml	Hedera helix extract	R05FB00	1 ml of syrup contains: 7 mg *Hedera helix folium* *extractum siccum* (5–7.5:1)	Over 1 year of age. For children under 1 year of age it must be prescribed by a doctor	OTC1	Early childhood	Upper respiratory tract, cough
27.	Prospan Liquid	7 mg/ml oral liquid—5 ml × 21	Hedera helix extract	R05FB00	1 ml of syrup contains: 7 mg *Hedera helix folium* *extractum siccum* (5–7.5:1)	Over 6 years of age	OTC1	Pre-school period	Upper respiratory tract, cough
28.	Sinupret	syrup × 50 ml, × 100 ml, × 200 ml, × 500 ml	Plant medicinal product	R05CA10	100 g of syrup contains: extract (1:11) from: ✔ *Gentianae radix*; ✔ *Primulae flos calycibus*; ✔ *Verbenae herba*; ✔ *Rumicis herba*; ✔ *Sambuci flos* (1:3:3:3:3)	Over 2 years of age	OTC2	Early childhood	Upper respiratory tract, inflammation of the paranasal sinuses
29.	Sinupret	coated tablets × 50; × 100; × 200; × 500	Rumicis herbae pulvis/Sambuci flos pulvis/Gentianae radix pulvis/Primulae flos pulvis/Verbenae herbae pulvis	R05CA10	1 coated tablet contains: ✔ *Gentianae radix pulv*.—6 mg; ✔ *Primulae flos cum calycibus pulv*.—18 mg; ✔ *Rumicis herba pulv*.—18 mg; ✔ *Sambuci flos pulv*.—18 mg; ✔ *Verbenae herba pulv*.—18 mg.	Over 6 years of age (not recommended for children under 6 years of age owing to the lack of sufficient evidence)	OTC2	School period (early school age)	Upper respiratory tract, inflammation of the paranasal sinuses
30.	Sinupret Forte	coated tablets × 20; × 50; × 100; × 500	Rumicis herbae pulvis/Sambuci flos pulvis/Gentianae radicis pulvis/Primulae flos pulvis/Verbenae herbae pulvis	R05CA10	1 coated tablet contains: ✔ *Gentianae radix pulv*.—12 mg; ✔ *Primulae flos cum calycibus pulv*.—36 mg; ✔ *Rumicis herba pulv*.—36 mg; ✔ *Sambuci flos pulv*.—36 mg; ✔ *Verbenae herba pulv*.—36 mg	Not recommended for children under 12 years of age owing to the lack of sufficient data	OTC2	School period (middle school age)	Upper respiratory tract, inflammation of the paranasal sinuses
31.	Tavipec	150 mg gastro-resistant capsules, soft × 20; × 30	Spicae aetheroleum	R05CA00	1 capsule contains: *Spicae* *aetheroleum*—150 mg	Over 12 years of age	OTC1	School period (middle school age)	Upper respiratory tract, expectorant
32.	Tuspan	7 mg/ml syrup × 120 ml	Ivy leaf dry extract	R05CA12	1 ml of syrup contains: 7 mg *extract from Hedera* *helix* L. (5–7.5:1)	Over 2 years of age (not recommended for children under 2 years of age owing to their oversensitivity to the active substance)	OTC1	Early childhood	Upper respiratory tract, expectorant
33.	Tussavit	7.53 g/7.53 g/100 g syrup 125 g; 250 g	Thymi folium extr./Plantaginis folium	R05CA00	100 g of syrup contains: ✔ 7.53 g *Thymi vulgaris herba/Thymi zygis herba extractum fluidum* (1:1); ✔ 7.53 g *Plantaginis lanceolatae folium extractum fluidum* (1:1)	Over 2 years of age (not recommended for children under 2 years of age owing to the content of alcohol)	OTC2	Early childhood	Upper respiratory tract, cough
34.	Umckalor	20 mg film-coated tablets × 15; × 30	Plant medicinal product	R05	1 coated tablet contains: 20 mg. dried *liquid extract from Pelargonii sidoidi radix* (1:8–10) (EPs 7630)	Over 6 years of age	OTC1	School period (early school age)	Respiratory tract, acute infections, bronchitis, sinusitis
35.	Umckalor	8 g/10 g oral drops, solution × 20 ml; 50 ml; × 100 ml	Pelargonii sidoidi radicis extractum liquidum	R05	10 g (= 9.75 ml) contains *Pelargonii sidoidi radix extr. fliudum* (1:8–10) (EPs 7630)	Over 1 year of age	OTC1	Early childhood	Respiratory tract, acute infections, bronchitis, sinusitis

## Data Availability

Data are contained within the article.

## Abbreviations

OTC1	Over-the-counter medicines containing only a plant component
OTC2	Over-the-counter medicines containing a combination of plant components
OTC3	Over-the-counter medicines containing a combination of a plant substance + a synthetic substance
OTC4	Over-the-counter medicines containing a derivative
OTC5	Over-the-counter medicines containing a combination of a derivative + a synthetic substance
OTC6	Over-the-counter medicines containing a combination of a plant substance + a derivative + a synthetic substance
BB12	Breastfeeding babies by the 28th day
BB28	Breastfeeding babies by the 12th month
Ech	Early childhood
PSchp	Pre-school period
Schp, early	School period which includes the early school age
Schp, middle	School period which includes the secondary school age
Adul	Adolescence
